# Variation in the *CACNB2* gene is associated with functional connectivity of the Hippocampus in bipolar disorder

**DOI:** 10.1186/s12888-019-2040-8

**Published:** 2019-02-11

**Authors:** Fang Liu, Xiaohong Gong, Xudong Yao, Lingling Cui, Zhiyang Yin, Chao Li, Yanqing Tang, Fei Wang

**Affiliations:** 1 Department of Psychiatry, First Affiliated Hospital, China Medical University, 155 Nanjing North Street, Shenyang, 110001 Liaoning China; 2 The Clinical Hospital of Chengdu Brain Science Institute, MOE Key Lab for Neuroinformation, University of Electronic Science and Technology of China, Chengdu, China; 3 A605, Building School of Life SCiences, Songhu Road 2005, Dinstric Yangpu, Shanghai, China; 4 Department of Radiology, First Affiliated Hospital, China Medical University, Shenyang, Liaoning People’s Republic of China; 5 Department of Geriatric Medicine, First Affiliated Hospital, China Medical University, Shenyang, Liaoning People’s Republic of China; 6 Brain Function Research Section, First Affiliated Hospital, China Medical University, Shenyang, Liaoning People’s Republic of China

**Keywords:** *CACNB2*, 11,013,860, Bipolar disorder, Resting-state functional connectivity (rs-FC)

## Abstract

**Background:**

Calcium voltage-gated channel auxiliary subunit β2 is a protein that, in humans, is encoded by the *CACNB2* gene. The β2 subunit is an auxiliary protein of voltage-gated calcium channels, which is predominantly expressed in hippocampal pyramidal neurons. A single-nucleotide polymorphism at the *CACNB2* gene (rs11013860) has been reported in genome-wide association studies to be associated with bipolar disorder (BD). However, the neural effects of rs11013860 expression are unknown. Thus, the current study investigated the mechanisms of how the *CACNB2* gene influences hippocampal-cortical limbic circuits in patients with bipolar disorder (BD).

**Methods:**

A total of 202 subjects were studied [69 BD patients and 133 healthy controls (HC)]. Participants agreed to undergo resting-state functional magnetic resonance imaging (rs-fMRI) and have blood drawn for genetic testing. Participants were found to belong to either a CC group homozygous for the C-allele (17 BD, 41 HC), or an A-carrier group carrying the high risk A-allele (AA/CA genotypes; 52 BD, 92 HC). Brain activity was assessed using resting-state functional connectivity (rs-FC) analyses.

**Results:**

A main effect of genotype showed that the rs-FC of the AA/CA group was elevated more than that of the CC-group between the hippocampus and the regions of right-inferior temporal, fusiform, and left-inferior occipital gyri. Additionally, a significant diagnosis × genotype interaction was noted between the hippocampus and right pars triangularis. Furthermore, in BD patients, the AA/CA group showed lower rs-FC when compared to that of the CC group. Additionally, individuals from HC within the AA/CA group showed higher rs-FC than that of the CC group. Finally, within C-allele-carrying groups, individuals with BD showed significantly increased rs-FC compared to that of HC.

**Conclusions:**

Our study demonstrates that BD patients with the *CACNB2* rs11013860 AA/CA genotype may exhibit altered hippocampal-cortical connectivity.

## Background

Mental illnesses are complex conditions that affect a substantial portion of the population. Primary symptoms include abnormalities in perception and cognition that result in behavioral and volitional impairments [1]. Bipolar disorder (BD) is a relatively common mental illness that is highly heritable, leading to the general belief that genes are an important risk factor [2]. The biomarker rs11013860 has been reported to be associated with BD. This biomarker is located in the *CACNB2* gene, which encodes for the β subunit of voltage-gated calcium channels (Ca_v_) and has been shown to be expressed in hippocampal pyramidal neurons in Han Chinese individuals [3]. The *CACNB2* gene, which is located at 10 p12, spanning an approximately 398.5 kb genomic region, consists of 14 exons. *CACNB2* encodes an auxiliary Ca_v_ subunit which interacts with L-type calcium-channel subunits to promote their trafficking to the plasma membrane, increase their function, and regulate their modulation by other signaling proteins and molecules [4, 5]. *CACNB2* variation provides a promising link for investigating the potential underlying molecular mechanisms of BD. One genome-wide association (GWAS) study of BD in the Han Chinese population found that rs11013860 in the *CACNB2* gene is associated with BD-I (*p* = 5.15*10^− 5^) [6]. A separate study conducted joint analyses of four markers (*CACNA1C* rs10848635, *CACNA1E* rs10848635, *CACNB2* rs11013860, and *CACNG2* rs 2,284,018) and found higher accumulative risk in individuals diagnosed with BD-I (*p*_trend_ = 0.006) and BD-II (*p*_trend_ = 0.017). Combined analysis with independent-replication samples further supported the association of rs11013860 with BP-I (*p* <0 .001) [7]. Accumulating evidence suggests that gene alterations of *CACNB2* in the hippocampus may cause changes to hippocampal circuitry, resulting in dysfunction of hippocampal neuroconnectivity similar to what is observed in BD. However, how rs11013860 may mediate this association is unknown.

Resting-state functional magnetic resonance imaging (rs-fMRI) is a promising tool for investigating the intrinsic functional connections among anatomically distinct brain regions within specific neural networks [8]. The intrinsic functional connectivity (FC) of hippocampal networks has been identified using rs-fMRI under both healthy and pathological conditions [9]. Further, genetic imaging technology has been utilized to explore the relationship between *CACNB2* gene polymorphisms and brain activity [10, 11], and to identify neural circuits linked to the genetic risk for heritable neuropsychiatric disorders [12]. As previous studies have confirmed that both the *CACNB2* gene is associated with BD, and that the *CACNB2* gene is distributed in hippocampal pyramidal neurons [3], the hippocampus was chosen as the region of interest (ROI) in the current study. We examined the hippocampus to identify any mediating effects of *CACNB2* rs11013860 on hippocampal-cortical connectivity in BD.

## Materials and methods

### Subjects

The current study was approved by the Ethics Committee of First Affiliated Hospital of China Medical University (Shenyang China). Each participant provided written informed consent prior to commencement of the study. If their age was less than 18-years-old, they and their parental/legal guardian also provided written informed consent. Sixty-nine BD patients (mean age = 27.19 ± 9.46 years, 68.12% female) and 133 healthy controls (mean age = 28.52 ± 8.07 years, 60.15% female) were recruited to undergo rs-fMRI and genetic testing. The BD patients were comprised of 31 BD-I (mean age = 29.58 ± 9.05 years, 64.52% female) and 38 BD-II (mean age = 25.66 ± 9.75 years, 71.05% female), which were recruited from the outpatient clinics of the Department of Psychiatry at First Affiliated Hospital of China Medical University (Shenyang China). Healthy control (HC) participants were recruited from Shenyang (China) using a community advertisement. All HC met the following criteria: (1) age between 13 and 59 years; (2) no history of neuropsychiatric or other severe diseases; (3) no history of head injury; (4) no MRI-scan limitations. All BD patients met criteria for BD diagnosis delineated in the Diagnostic and Statistical Manual of Mental Disorders, Fourth Edition (known as the DSM-IV) according to a diagnostic assessment which fulfilled guidelines in the Structured Clinical Interview for DSM-IV, Patient Edition. Diagnosis was determined by two experienced clinical psychiatrists. Exclusion criteria included the presence of the following: (1) any history of major bodily disease; (2) any history of moderate or severe head injury, head trauma, neurologic disorder, or mental retardation; (3) alcohol or substance abuse or dependence; and/or (4) the presence of a concurrent and major physical illness that could lead to mood disorder symptoms. The control subjects were interviewed using the Structured Clinical Interview for the DSM-IV, Non-patient edition (SCID-I/NP) to ensure no presence of BD or other psychiatric disorders.

### Extraction and genotyping

Ten-milliliter blood samples were collected from participants for DNA extraction. Genotypes of single nucleotide polymorphisms (SNPs) at rs11013860 were determined using the Sanger sequencing method. Subjects were further divided into two groups: a CC-allele group (CC genotypes; 17 BD, 41 HC; mean age = 29.14 ± 8.35 years, 67.24% female) and risk A-carrier group (AA/CA genotypes; 52 BD, 92 HC; mean age = 27.88 ± 8.68 years, 61.97% female). Minor allele frequencies were 0.45%. Genotype frequencies were consistent with Hardy-Weinberg equilibrium (HWE) expectations (BD: *χ*^2^ = 1.24, *p* =0 .26; HC: *χ*^2^ = 0.21, *p* = 0.65).

### fMRI data acquisition

A GE Signa HDX (U.S.A.) 3.0 T MRI scanner at the First Affiliated Hospital of China Medical University (Shenyang, China) was used to acquire rs-fMRI data. Head motion was minimized with restraining foam pads. Soft pads and earplugs were used during scanning to restrict head motion and reduce scanner noise. Participants were asked to keep their eyes closed, but remain awake during the scan. A gradient-echo planar imaging (EPI) sequence parallel to the AC-PC plane was used to obtain rs-fMRI images, with the following scan parameters: repetition time (TR) = 2000 ms; echo time (TE) = 40 ms; image matrix = 64 × 64; field of view (FOV) = 24 × 24 cm^2^; 35 contiguous slices of 3 mm without gap; scan time = 6 min 40 s.

### Image analysis and processing

Preprocessing of rs-fMRI data was conducted using SPM12 (www.fil.ion.ucl.ac.uk/spm/software/spm12) and the rs-fMRI processing and analysis toolbox (DPABI; http://rfmri.org/dpabi). The first 10 images were deleted due to magnetic saturation effects. The remaining data were then further preprocessed, which included slice-timing correction, head-motion correction, spatial normalization, and smoothing. Head motion parameters were computed by estimating translation in each direction and angular rotation about each axis for each volume. According to the record of head motion within each rs-fMRI run, participants were excluded if their head motion was > 2.5 mm maximal displacement in any of the x, y, or z directions, or 2.5° of any angular motion throughout the course of the scan [13]. Subsequent data preprocessing included removal of linear trends and temporal filtering (band pass, 0.01–0.08 Hz) to reduce the effects of low-frequency drift and high-frequency noise. Linear regression of head motion parameters, global mean signal, white matter signal, and cerebrospinal fluid signal were performed to remove effects of these covariates.

### ROI-based functional connectivity analysis

The bilateral hippocampus ROI was defined according to the automated anatomical labeling (AAL) template [14]. For each subject, the ROI time series was extracted as the mean time series across all voxels within that region [15]. The time series of each ROI was correlated with those of the whole brain. The Pearson correlational coefficient (*r*) was obtained by the voxel-voxel method. Finally, normality of the data was improved by converting *r* values to *z* values through Fisher’s *r*-to-*z* transformation.

### Statistical analysis

Demographic data (sex, age, education) were analyzed using *t-*tests with diagnostic groups (BD, HC) and genotype groups (CC, AA/CA) as between-subject factors. An analysis of variance (ANOVA) was used to compare different genotypes between the BD and HC groups. All statistical analyses were performed using SPSS 22.0 software.

A voxel wise ANOVA (2 × 2 ANOVA: diagnosis × genotypes) was used to determine the effects of diagnosis and genotype on FC, and age and sex were considered covariates. A post hoc *t*-test was used to explore main effects and interactions. The contrast map threshold was set at *p* < 0.01 for each voxel, with a cluster size of at least 49 voxels (1323 mm^3^), which was equal to the corrected threshold of *p* < 0.05, as determined by Gaussian random field (GRF).

## Results

### Participant characteristics

There were no significant effects for age or sex among BD and HC participants (Table 1).

**Table 1:** Demographic and clinical data of participants

Characteristics	Genotype	HC (*n* = 133)	BD (*n =* 69)	*t*/χ^2^	*p* value
Age (years)	AA	29.79 ± 9.16	28.23 ± 7.68	1.04	0.30
	CA	28.14 ± 8.21	25.62 ± 9.44		
	CC	28.39 ± 7.25	30.94 ± 10.60		
Sex (M:F)	AA	11:13	4:9	1.29	0.26
	CA	28:40	13:26		
	CC	14:27	5:12		
Education level (years)		14.92 ± 3.40	13.03 ± 3.11	–	–
Handedness (Right/Left/Both)		127/5/1	66/2/1	–	–
Medication, yes		–	52(75.36%)		
Anti-depressants		–	25(36.23%)		
Antipsychotics		–	30(43.48%)		
Mood stabilizer		–	48(57.97%)		
HWE	χ^2^	0.21	1.24	–	–
	*p* value	0.65	0.26	–	–
Allele	A-allele	116 (0.44)	65 (0.47)	–	–
	C-allele	150 (0.56)	73 (0.53)	–	–

Data are presented as mean ± SD (Standard deviation)

### Resting-state functional connectivity (rs-FC)

Influence of diagnosis and genotype on rs-FC between CC and AA/CA genetic subgroups in BD and HC are listed in Table 2.

**Table 2:** Clusters exhibiting the influence of group and genotype on the values of FC between CC and CA/AA genetic subgroups in BD and HC

Brain area	Cluster size	Peak MNI coordinates			Peak F value
		X	Y	Z	
Main effect of genotypes					
right inferior temporal/right fusiform gyri	80	48	−57	−18	12.07
left fusiform/left inferior occipital gyri	50	−39	−72	−15	23.25
Diagnostic groups × genotype interaction					
right pars triangularis	128	54	33	9	19.31

These findings correspond to a corrected *p* <0 .01 by GRF correction. Cluster size is in mm^3^

**Fig. 1:**
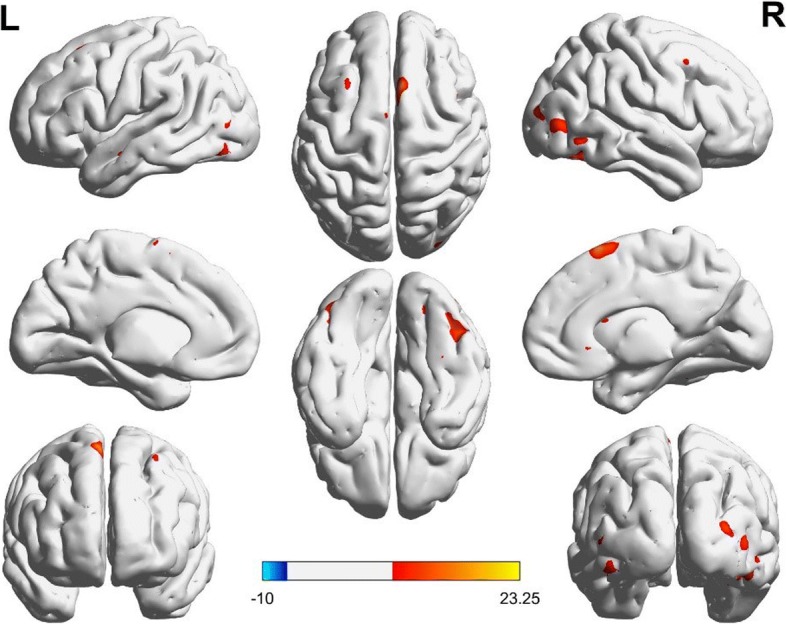
Main effect of genotype on the rs-FC between AA/CA group and CC group. Clusters presenting lower (blue) or higher (red) rs-FC, *p* < 0.01 GRF-corrected

**Fig. 2:**
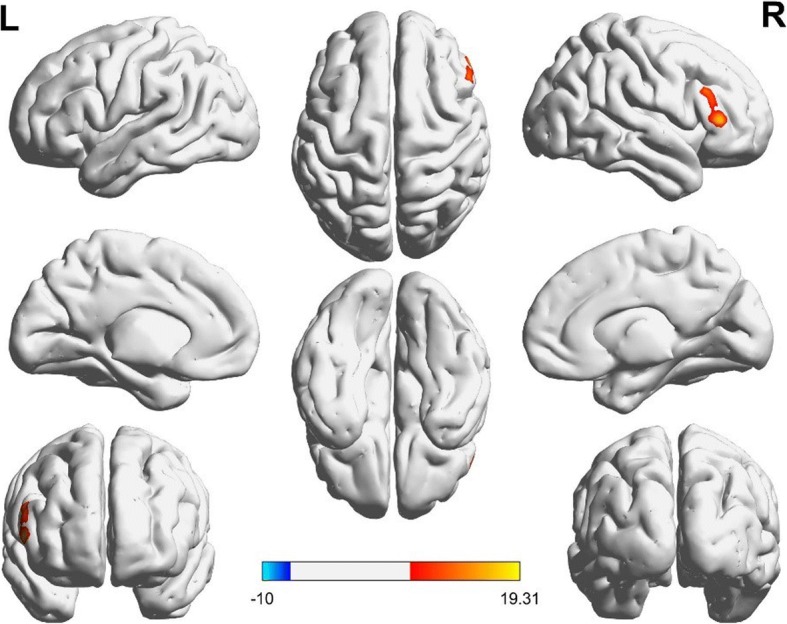
Interaction between rs11013860 genotype and diagnosis in BD and HC. Clusters presenting lower (blue) or higher (red) rs-FC, *p* < 0.01 GRF-corrected

**Fig. 3:**
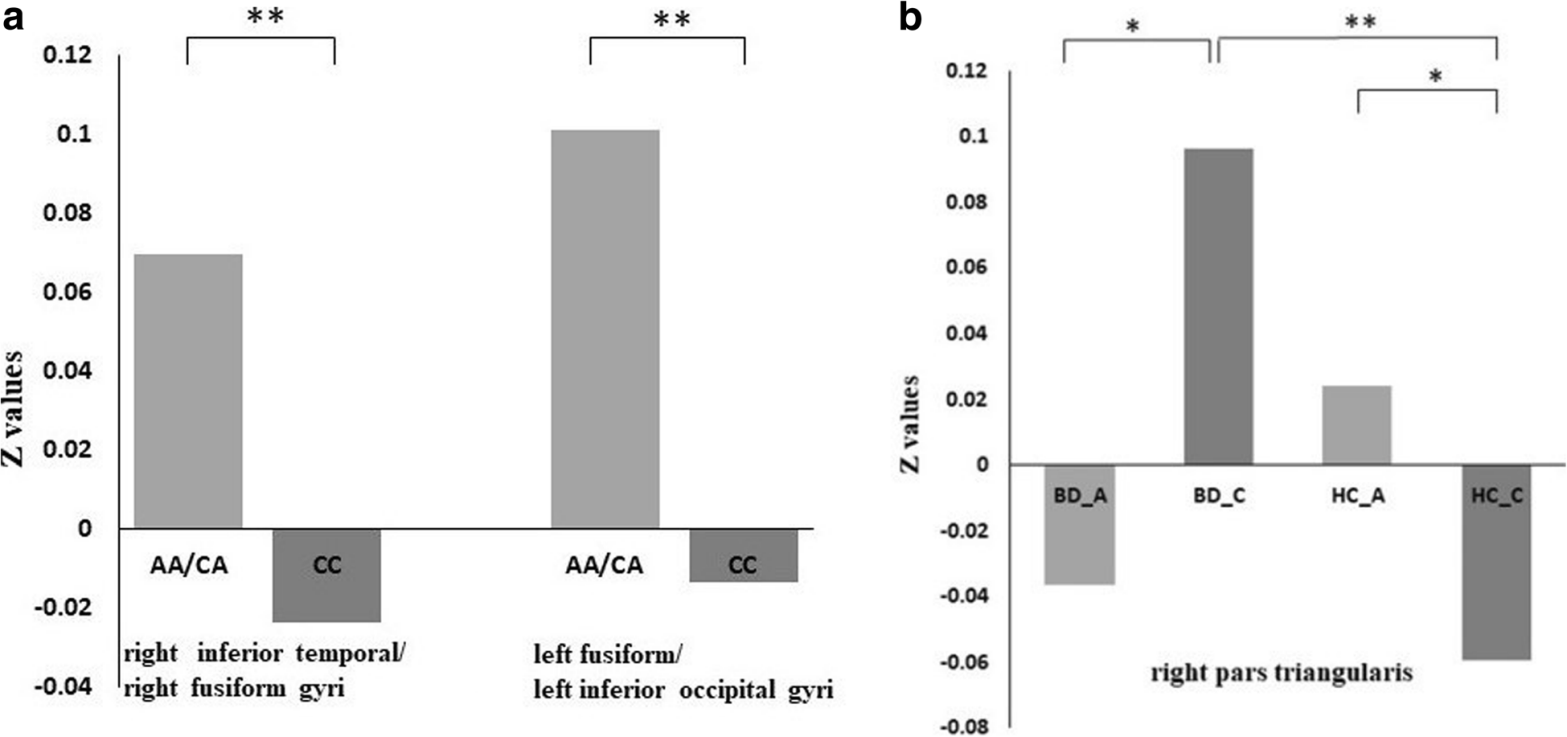
**a** Main effect of genotype showing that carriers of high-risk A-allele exhibited higher rs-FC than carriers of C- between the hippocampus and the regions of right inferior temporal, fusiform, and left inferior occipital gyri (independent *t*-test, *p* <0 .001). **b** Interaction graph showing that in BD patients rs-FC was decreased between the hippocampus and right pars triangularis in A-allele carrying individuals compared to that of C-allele carrying individuals, in HC the AA/CA group showed higher rs-FC than CC group, and in carrying C-allele groups the patients with BD had significantly increased rs-FC compared to that of HC

After performing a one-way ANOVA on the FC maps, the main effect of diagnosis was not statistically significant. However, there was a significant main effect of genotype between the hippocampus and the regions of the right-inferior temporal and right fusiform gyri (*t* = 3.91 *p* < 0.001, corrected) and left fusiform/left inferior occipital gyri (*t* = 4.11 *p* < 0.001, corrected; Fig. 1). Individuals carrying high-risk A-allele exhibited higher rs-FC than individuals carrying the C-allele (Fig. 3a). Additionally, there was a significant interaction of genotype-by-diagnosis between the hippocampus and right pars triangularis (F= 7.08, *p* < 0.001, corrected; Fig. 2). Further, in BD patients, the AA/CA group showed lower rs-FC than that of the CC group. Additionally, in individuals with HC, the AA/CA group showed higher rs-FC than that of the CC group. Finally, in C-allele-carrying groups, the patients with BD had significantly increased rs-FC compared to that of the CC group (Fig. 3b).

## Discussion

L-type calcium channels (LTCCs) are widely expressed in the central nervous system, and have been shown to influence neurotransmitter release and neuronal excitability [16]. Studies have shown that altered calcium levels in cells might affect vulnerability (i.e. impaired resilience) observed in neurons of patients with BD [6, 17]. LTCCs are formed by the principal χ1 subunits with auxiliary χ2δ and cytosolic β, γ subunits [3]. The β subunit is coded by *CACNB2* genes, which are distributed only in hippocampal pyramidal, thalamic, and cerebellar Purkinje cells in the brain [3, 18, 19].

In the last few years, both GWAS and Integrated Pathway-Based Studies have identified associations between SNPs in *CACNB2* and serious mental disorders, including BD [6, 7, 20]. A study by Soldatov found that CACNB2 modulated Cav1.2 channels and showed decreased and incomplete inactivation of calcium currents, possibly causing neuronal dysregulation related to the parthenogenesis of BD [21]. More recent evidence suggests that the alteration of *CACNB2* rs11013860 may be a molecular mechanism of genetic risk in BD [6].

In our study, we did not observe abnormal neural circuits between BD and HC. This may be related to sample size, medication, and/or disease status. However, genotype was found to be associated with neural connectivity between the hippocampus and the regions of right inferior temporal, fusiform, and left inferior occipital gyri. Individuals carrying the high-risk A-allele exhibited higher rs-FC than individuals carrying the C-allele. Early research has confirmed that there are cellular connections from the temporal gyrus to the hippocampal area [22], and that this region is associated with processing memory, as well as visual and linguistic information. It is possible that carrying this risk gene may result in impairment of these functions. The fusiform gyrus is related to positive task activation in cognitive behaviors, such as object, face, body, and character recognition [23–25]. Additionally, the inferior occipital gyrus has been shown to be related to association and recognition memory [26, 27]. The increased excitability of the A-allele risk gene in the neural circuitry between these brain regions and the hippocampus suggests that carrying the risk gene could lead to dysfunction, possibly impairing social function and memory.

Additionally, the current study observed significant interactions between rs11013860 genotypes and diagnoses between BD and HC groups within the neural circuits of the hippocampus and right pars triangularis. In BD patients carrying the A- risk gene, rs-FC was decreased when compared to that of patients carrying the C-allele. In C-allele-carrying groups, patients with BD had significantly increased rs-FC compared to that of HC. Our findings suggest that the excitability of the neural circuitry underlying the condition of gene-disease interactions may have different results. And the potential abnormalities of the neural circuits between the hippocampus and right pars triangularis in BD patients with the A- risk group suggest that the risk A allele is associated with frontal-hippocampal cortical dysfunction activity in BD.

The pars triangularis is involved in a specific type of language processing and plays a role in the cognitive control of memory [28–30], and is a part of the prefrontal cortex. Anatomical studies have shown that there are complex neural projections between the hippocampus and the prefrontal cortex [31, 32], which participate in a variety of cognitive functions and are associated with the occurrence of a variety of mental disorders [22, 23, 33]. Our study showed impaired prefrontal-hippocampal cortical neuroconnectivity in patients with BD that also carried the A allele. Previous studies have found that dysregulation of Ca^2+^ signaling pathways has been implicated in the development of BD [24]. In patients with BD, voltage-gated Ca^2+^ channels can be activated by membrane depolarization, resulting in increased neuronal excitability [25]. The CACNB2 results in the changing of channel activity by encoding the Ca^2+^ channel β-subunit, regulating the intensity of current of the Ca^2+^ channel, and controlling the membrane characterization of the χ1 subunit [3, 21]. Therefore, it is possible that the interaction between gene and disease brings the neural circuitry between hippocampus and right pars triangularis back to normal.

However, it remains unknown when and how this interaction could change the neural circuitry in the brain. Many factors could influence the occurrence and development of BD, which have been well established as risk factors for the occurrence of disease [26, 27].

## Conclusion

In summary, the present study demonstrated, for the first time, that *CACNB2* rs11013860 AA/CA genotype may affect regional brain activity in BD patients. This suggests that the influence of *CACNB2* variation may be one mechanism that contributes to the neural circuitry of BD.

## Abbreviations

AA/CA: high-risk genetic subgroup
AAL: automated anatomical labeling
BD: Bipolar disorder
Cav: voltage-gated calcium channels
CC: allele genetic subgroup
FC: functional connectivity
GWA: genome-wide association
HC: Healthy controls
HWE: Hardy-Weinberg equilibrium
LTCC: L-type calcium channels
ROI: region of interest
rs-FC: resting-state functional connectivity
rs-fMRI: resting-state functional magnetic resonance imaging
